# Walkthrough Sanitization Gates for COVID-19: A Preventive Measure or Public Health Concern?

**DOI:** 10.4269/ajtmh.20-0533

**Published:** 2020-06-03

**Authors:** Tauqeer Hussain Mallhi, Yusra Habib Khan, Nasser Hadal Alotaibi, Abdulaziz Ibrahim Alzarea

**Affiliations:** Department of Clinical Pharmacy, College of Pharmacy, Jouf University, Al-Jouf, Sakaka, Kingdom of Saudi Arabia

## Abstract

As the COVID-19 pandemic continues to gain momentum around the world, several measures are being put in place to control its spread. One such effort includes the installation of walkthrough sanitization gates to disinfect passersby and prevent cross infection. However, there is lack of clinical evidence on the effectiveness of these walkthrough gates to contain COVID-19. Moreover, there are potential public health concerns associated with these walkthrough gates. Spraying individuals with disinfectant chemicals is strongly discouraged by various health authorities around the globe because of their propensity for eye and skin irritation, bronchospasm following inhalation, and gastrointestinal effects such as nausea and vomiting. This article underscores that the risks associated with the use of these walkthrough gates overweigh any potential benefits. Health authorities must discourage their use and should focus efforts on other preventive measures such as social distancing, wearing masks, and hand hygiene to prevent the spread of COVID-19 among the general public.

## BACKGROUND AND RECOMMENDATION

The COVID-19 pandemic has become a global challenge transcending territorial, political, ideological, religious, cultural, and academic boundaries. The containment of the disease is of utmost priority across the world. Because an effective vaccine or specific treatments are currently unavailable, disinfection, sanitization, and distancing are considered the most appropriate measures to mitigate virus transmission. Health organizations around the globe have proposed various precautionary maneuvers to combat the disease, including physical distancing, forced or self-quarantine, and personal hygiene. As the COVID-19 pandemic continues to gain momentum around the world, several measures are being put in place to control its spread. One such effort includes the installation of walkthrough sanitization gates to disinfect passersby and prevent cross infection within the premises. These gates, also termed as sanitizing or disinfectant chambers, tunnels, boxes, booths, or partitions, provide an overhead shower of disinfectant solution for 20–30 seconds as individuals pass through them. Currently, various vendors are actively promoting them, with the claim of killing 99.9% of coronavirus. They have recently been installed in various countries at public places, shopping malls, supermarkets, mosques, railway stations, hospitals, offices, and factories.^1^ Demand for these walkthrough gates has sharply surged amid relaxation of the lockdown in various countries.

In an extensive search (through various search engines using relevant key words along with Boolean operators), we did not come across any scientific evidence regarding the effectiveness of walkthrough gates for the prevention of COVID-19. These walkthrough gates use diluted disinfectant solutions of chlorine, bleach (sodium hypochlorite), alcohol, or chloroxylenol.^13^ Spraying or fogging of certain chemicals, such as formaldehyde, chlorine-based agents, or quaternary ammonium compounds, is not recommended because of adverse health effects. Moreover, the WHO has endorsed that spraying individuals with disinfectants is not recommended under any circumstances. This could be physically and psychologically harmful, and will not reduce an infected person’s ability to spread the virus through droplets or contact. Moreover, spraying individuals with chlorine and other toxic chemicals could result in eye and skin irritation, bronchospasm due to inhalation, and gastrointestinal effects such as nausea and vomiting.^4^ During Ebola outbreaks, disinfectant intoxication has been observed among healthcare providers and Ebola survivors; deliberate exposure of humans to chlorine spray was associated with pulmonary, dermatological, and ophthalmic complications even with the use of personal protective equipment, and even after the first exposure to chlorine spray.^5^ Disinfectants in the forms of fumes and aerosols have high propensity to ingress mucus membrane, posing immediate respiratory symptoms and dermal irritation. In addition, mixing of bleach with other disinfectants may have disastrous effects. The combination of bleach and alcohol can lead to the formation of chloroform, which is a probable carcinogen.^3^ Fumigation is meant for inanimate objects and surfaces, and it should never be used on people.

Although several health authorities around the globe have issued advisories against the use of walkthrough gates,^6^ however, their popularity is increasing in various countries.^7,8^ The use of these unstandardized gates violates WHO recommendations. In addition, these gates and tunnels may provide a false sense of protection, and attention to this approach could undermine evidence-based coronavirus preventive measures such as adequate hand hygiene, high-touch surface disinfection, and physical distancing. We believe that these walkthrough gates have substantial safety concerns and pose serious public health hazards. We urge health authorities around the globe to discourage the use of walkthrough gates amid the lack of clinical evidences and rather to emphasize the importance of social distancing, wearing masks, and hand hygiene to prevent the spread of COVID-19 among the general public.

**Figure 1.**
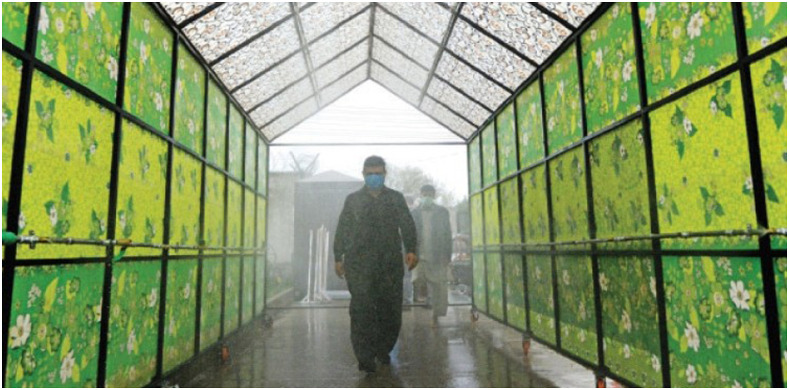
Disinfectant walkthrough tunnel installed in Islamabad, Pakistan; Source: https://arynews.tv/en/disinfecting-walk-through-gates-cant-kill-covid-19/.

## References

[1] Khan A, 2020 Fad or future?: Experts question efficacy of disinfection gates. The Express Tribune.

[2] CodeBlue, 2020 MOH Kills Virus Disinfection Chamber Proposals. CodeBlue - Health Is Human Right.

[3] Zaidi SK, 2020 How Effective Are Walk-Through Disinfection Gates against Covid-19? the Express Tribune (Blogs).

[4] WHO, 2020 Cleaning and Disinfection of Environmental Surfaces in the Context of COVID-19: Interim Guidance, 15 May 2020. Geneva, Switzerland: World Health Organization.

[5] Mehtar S Bulabula AN Nyandemoh H Jambawai S, 2016 Deliberate exposure of humans to chlorine-the aftermath of Ebola in west Africa. Antimicrob Resist Infect Control 5: 45. 27895903 10.1186/s13756-016-0144-1 PMC5109677

[6] Reporter, 2020 Covid-19: Morocco Bans Walk-Through Disinfectant Gates. The North Africa Post.

[7] CGTN R, 2020 Walk-Through Disinfectant Tunnel installed in Pakistan. CGTN.

[8] Shouk AA, 2020 COVID-19 Security: How Will Dubai Malls Control Crowds, Monitor Temperature? Gulf News.

